# Neuronal and glial vulnerability of the suprachiasmatic nucleus in tauopathies: evidence from human studies and animal models

**DOI:** 10.1186/s13024-023-00695-4

**Published:** 2024-01-10

**Authors:** Gowoon Son, Thomas C. Neylan, Lea T. Grinberg

**Affiliations:** 1 Memory and Aging Center, Weill Institute for Neurosciences, Department of Neurology, University of California, San Francisco, San Francisco, CA USA; 2 Department of Psychiatry, University of California, San Francisco, San Francisco, CA USA; 3 Global Brain Health Institute, University of California, San Francisco, San Francisco, CA USA; 4 Department of Pathology, University of Sao Paulo Medical School, Sao Paulo, Brazil; 5 Department of Pathology, University of California, San Francisco, San Francisco, CA USA

**Keywords:** “Alzheimer’s disease”, “Progressive supranuclear palsy”, “Neurodegenerative disease”, “Suprachiasmatic nucleus”, “Tau”, “Amyloid”, “Circadian dysregulation”, “Circadian clock”, “Astrocyte”, “Microglia”

## Abstract

Tauopathies, a group of neurodegenerative diseases that includes Alzheimer’s disease, commonly lead to disturbances in sleep-wake patterns and circadian rhythm disorders. The circadian rhythm, a recurring 24-hour cycle governing human biological activity, is regulated by the hypothalamic suprachiasmatic nucleus (SCN) and endogenous transcriptional-translational feedback loops. Surprisingly, little attention has been given to investigating tauopathy-driven neuropathology in the SCN and the repercussions of SCN and circadian gene dysfunction in the human brain affected by tauopathies. This review aims to provide an overview of the current literature on the vulnerability of the SCN in tauopathies in humans. Emphasis is placed on elucidating the neuronal and glial changes contributing to the widespread disruption of the molecular circadian clock. Furthermore, this review identifies areas of knowledge requiring further investigation.

Sleep-wake dyshomeostasis and circadian disturbances are among the most distressing symptoms in Alzheimer’s disease (AD) and non-AD tauopathies, often leading to significant medical, health, and institutionalization [1, 2]. Therefore, understanding the neuronal and molecular basis of the association between tau-driven neurodegeneration and the neuronal system regulating sleep-wake-circadian systems is critical for developing tailored treatments to mitigate these symptoms. Over recent years, a handful of studies have delved into the neural foundations of sleep-wake dyshomeostasis within specific human tauopathies. Despite the suprachiasmatic nucleus (SCN) being the master brain clock, little attention has been given to investigating tauopathy-related neuropathology in the SCN and the repercussions of SCN degeneration and circadian gene dysfunction in the human brains affected by tauopathies. This review summarizes the existing literature evidencing the vulnerability of the SCN in human tauopathies. Given the current understanding of circadian dysfunction within these conditions, we offer potential avenues for future research. Emphasis is placed on elucidating the neuronal and glial changes contributing to the widespread disruption of the molecular circadian clock. Furthermore, this review explores the reciprocal influences of gliosis and immune response within the SCN that can concomitantly contribute to exacerbating tauopathies.

For this review, searched the literature for the following terms: “Alzheimer’s disease”, “Progressive supranuclear palsy”, “Neurodegenerative disease”, “Sleep-wake disturbance”, “Pathology”, “Tau”, “Amyloid”, “Suprachiasmatic nucleus”, “Circadian rhythm”, “Circadian gene”, “Glia”, “Astrocyte”, “Microglia”, and “Inflammation”. Most of the papers found have been published in the last 5 years, underscoring the recent and substantial surge in research interest within this field.

## Tauopathies

**Fig. 1:**
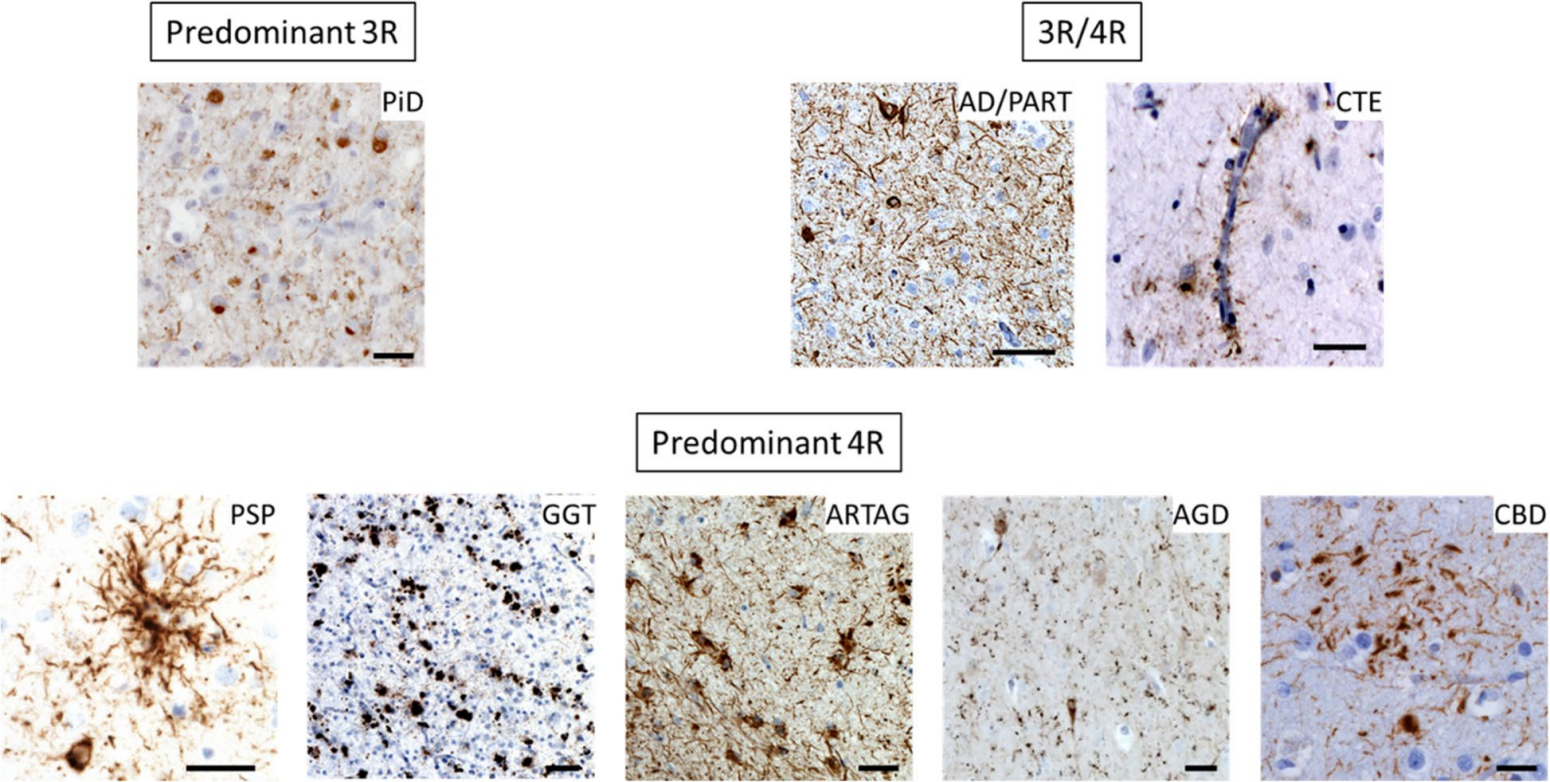
Pathological classification of tauopathies. Tau immunoreactivity in 3R, 4R, and 3R/4R tauopathies. Abbreviations: 3R: Three microtubule-binding repeats, 4R: Four microtubule-binding repeats, 3R/4R: A combination of three and four microtubule-binding repeats, PiD: Pick’s disease, AD: Alzheimer’s disease, PART: Primary age-related tauopathy, CTE: Chronic traumatic encephalopathy, PSP: Progressive supranuclear palsy, GGT: Globular glial tauopathy, ARTAG: Aging-related tau astrogliopathy, AGD: Argyrophilic grain disease, CBD: Corticobasal degeneration. Scale bar: 10 *μm*

Tauopathy is an umbrella term encompassing more than 20 well-defined, progressive neurodegenerative entities characterized by abnormal accumulation of protein tau in neurons and glial cells [3, 4]. Sporadic tauopathies are classified based on the pattern of morphological distribution of the inclusions, what types of cells accumulate tau, and the biochemical composition of tau inclusions, namely predominance of three microtubule-binding repeat (3R) tau, four microtubule-binding repeat (4R) tau or a combination of both (3R/4R) [3] (Fig. 1). Examples of 3R/4R tauopathies, include AD, chronic traumatic encephalopathy (CTE). Due to the absence of a consensus regarding whether primary age-related tauopathy (PART) should be classified as a subtype of Alzheimer’s disease (AD) or considered independently, we have incorporated PART into our literature search independently [5, 6]. Pick’s disease (PiD) falls into the 3R category. Progressive supranuclear palsy (PSP), corticobasal degeneration (CBD), globular glial tauopathy (GGT), argyrophilic grain disease (AGD), and aging-related tau astrogliopathy (ARTAG) belong to the 4R category [3]. Familial tauopathies exhibit distinct clinicopathological phenotypes depending on the specific microtubule-associated protein tau (MAPT) mutation [4, 7].

## Sleep-wake disorders in tauopathies

The control of sleep and wake behavior involves the interaction of two processes, sleep homeostasis and circadian rhythm (also known as processes S and C, respectively) [8]. Sleep homeostasis involves the gradual increase of sleep pressure that develops as a function of successive hours of being awake. Sleep behavior dissipates the accrued sleep pressure derived from wakeful activity and reduces the pathological impact of prolonged wake on the performance of the neuronal system. The circadian rhythm regulates a biological clock, a daily cycle of an alerting signal, that maintains wakefulness during the day while sleep readiness progressively grows, and then recedes in diurnal species when darkness falls, permitting sleep to naturally occur at night. Process C is entrained to the light-dark cycle and other environmental cues called Zeitgebers. Therefore, a rhythmic balance of two processes (interaction) results in a regular sleep-wake cycle entrained in the light-dark cycle. Circadian rhythm disorders can produce periods of abnormal sleepiness and wakefulness across the 24-hour day. This review is particularly focused on the neuronal basis of process C dysfunction in tauopathies. While sleep-wake homeostasis and circadian functions have been examined only in a limited number of tauopathies, the studies conducted have provided evidence of dysfunction, occasionally manifesting as early-stage symptoms (Table 1) [24–26]. Notably, these disturbances show distinctive phenotypes in the different tauopathies. For instance, PSP features short sleep duration, decreased rapid eye movement (REM) sleep and slow wave sleep (SWS), daytime hyperarousal, and reduced sleep drive [24, 27, 28], a unique pattern among neurodegenerative disorders. Sleep/wake dyshomeostasis in AD involves increasing nocturnal awakenings, normal sleep duration, a prominent decrease in SWS [29–33], and a propensity for daytime sleep as measured by the Multiple Sleep Latency Test [25, 31, 34, 35]. A limited number of studies have delved into the examination of sleep-wake patterns in corticobasal syndrome, serving as a proxy for CBD. The findings from these studies have presented conflicting results, with some indicating only slight distinctions compared to control groups, while others suggest a pattern resembling what is observed in AD. In certain cases, there have even been indications of the potential presence of REM behavior disorder [20, 36]. Unfortunately, only one-third of corticobasal syndrome cases exhibit CBD pathology [37], and the majority of these studies did not involve cases with postmortem examinations, making it challenging to arrive at definitive conclusions. A neuropathological study with autopsy confirmed CBD cases show preservation of wake-promoting neurons [38]. Although more systematic studies are needed, CTE is associated with REM sleep disorder [39]. In recent years, a few studies have begun to uncover insights into tau-associated neurodegeneration affecting the brain’s nuclei responsible for regulating sleep and wake patterns [38, 40]. While these studies remain relatively scarce and have been limited in scope due to the complexity of conducting such research in humans, they have revealed a distinct pattern of neurodegeneration linked to specific tauopathies that starts to inform therapeutic strategies [28, 41].

**Table 1:** Evidence from clinical and neuronal investigations of sleep-wake dyshomeostasis and circadian dysregulations in pathologically confirmed tauopathies

Tauopathies (confirmed pathologically) with sleep-wake dyshomeostasis and circadian dysregulations		Evidence of sleep-wake-circadian disturbances in clinical records	Investigation of the neuronal basis of sleep-wake disturbances ^a)^	Reference ^a)^
3R + 4R	Alzheimer’s disease (AD)	Yes	Yes	[9–15]
	Chronic traumatic encephalopathy (CTE)	Yes	Yes	[16–18]
	Postencephalitic parkinsonism	Yes ^b)^	Not tested	[19]
	Primary age-related tauopathy (PART)	Not tested	Not tested	
4R	Progressive supranuclear palsy (PSP)	Yes	Yes	[20, 21]
	Corticobasal degeneration (CBD)	Yes	Yes	[11, 20]
	Argyrophilic grain disease (AGD)	No	Not tested	
	Aging-related tau astrogliopathy (ARTAG)	No	Not tested	
	Globular oligodendroglial inclusions (GGT)	Not tested	Not tested	
3R	Pick’s disease (PiD)	Yes	Not tested	[22, 23]

a) Specifically for involvement of sleep/wake-promoting nuclei or SCN

b) A case study (1 case)

## Circadian dysfunction in tauopathies

While it is a challenging task to distinguish circadian function from sleep-wake homeostasis due to their intricate interconnection, research exploring disruptions in circadian rhythms, such as alterations in period, phase, and amplitude of rest/active brain activity as well as automatic physical responses (*e.g.*, body temperature, heart rate, and respiratory rate and hormone levels in plasma/saliva (*e.g.*, melatonin and corticotropin-releasing hormone), has indicated the existence of circadian dysfunction in the few tauopathies tested (*i.e.* AD and PSP) [42–45]. *Winer* and colleagues compared actigraphy data with tau-PET and amyloid-PET tracer retention and found an association between tau burden (but not amyloid burden) and sleep fragmentation [46]. PSP patients exhibit worsened circadian activity rhythms, including a decrease in *amplitude* and *mesor* compared to the age-matched control [47]. In studies with a limited number of patients and controls, individuals with PSP showed a significant increase in nighttime core-body temperature than those with Parkinson’s disease, a synucleinopathy [48], and a higher nighttime blood pressure compared to controls [49]. Higher tau-PET tracer retention was correlated with lower daytime body temperature in a cognitively normal adult [50]. In summary, despite the limited number of studies and methodological constraints, there is confluent evidence of circadian dysfunction in tauopathies. This evidence points to a disease-specific circadian dysfunction pattern that warrants a thorough exploration of the underlying mechanisms. For instance, this could involve mapping the scope and repercussions of tauopathies within the human SCN, which serves as the central circadian clock.

## The suprachiasmatic nucleus and regulation of the circadian rhythm

**Fig. 2:**
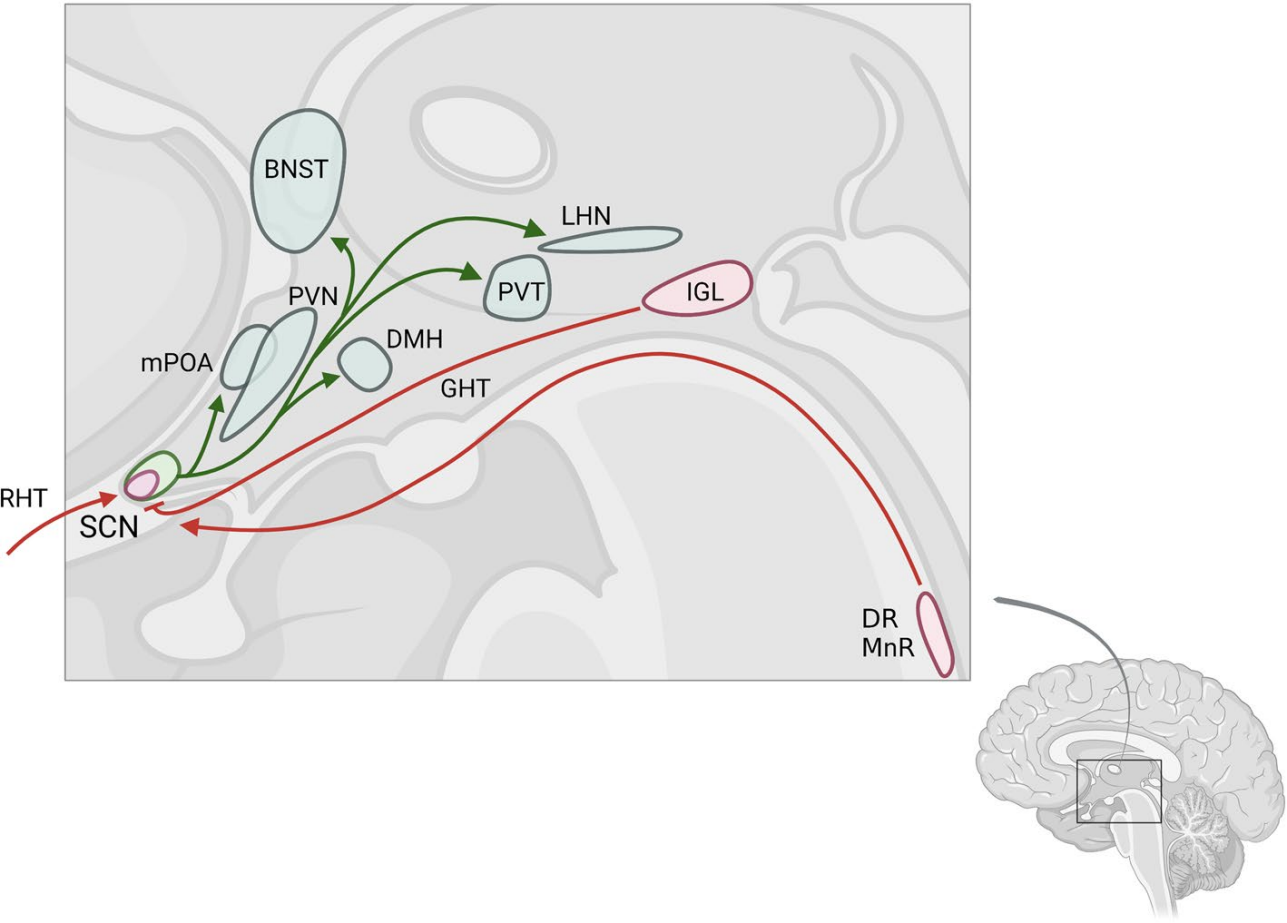
The suprachiasmatic nucleus circuitry, including its monosynaptically connected regions. Scheme of the human brain depicting the SCN and its monosynaptic afferent (in red) and efferent (in green) projections. Abbreviations: BNST: Bed nucleus of the stria terminalis, DR, MnR: Dorsal and median raphe nucleus, DMH: Dorsomedial hypothalamic nucleus, GHT: Geniculohypothalamic tract, IGL: Thalamic intergeniculate leaflet, LHN: Lateral habenula nucleus in thalamus, mPOA: Medial preoptic area, PVN: paraventricular nucleus of the hypothalamus, PVT: Paraventricular thalamic nucleus, RHT: Retinohypothalamic tract, SCN: Suprachiasmatic nucleus

The human SCN are bilateral structures of approximately 50,000 neurons each, located in the anterior hypothalamus [9]. The SCN acts as the central circadian pacemaker, playing a vital role in regulating and synchronizing circadian rhythms and cellular sleep-wake cycles through direct connections to various hypothalamic nuclei and subcortical regions (Fig. 2). SCN integrity is crucial for maintaining sleep homeostasis and circadian clock rhythmic within the sleep-wake regulatory network [51].

**Fig. 3:**
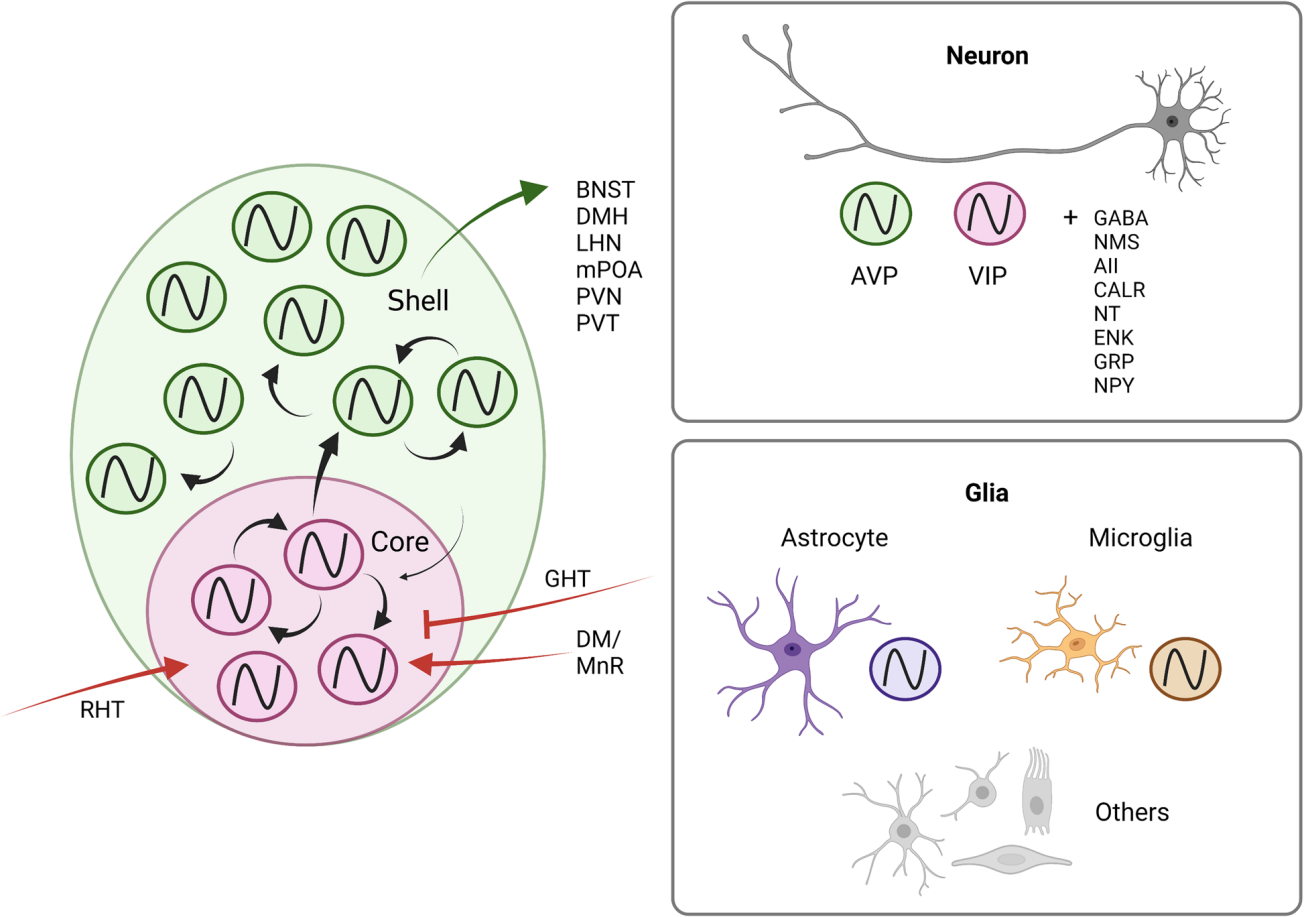
The topographical organization of the SCN. More than ten distinct neuronal populations have been identified in the suprachiasmatic nucleus (SCN), but the primary neuronal populations responsible for regulating circadian rhythms are AVP (arginine vasopressin) and VIP (vasoactive intestinal peptide) neurons. AVP neurons (green shade) and VIP neurons (red shade) are topographically arranged in the shell and core regions of the SCN, respectively. The black arrows indicate the intercellular projection of circadian rhythms, with a stronger signal transmission from the core to the shell. Furthermore, astrocytes and microglia are present throughout the SCN, and emerging evidence highlights their significant roles in regulating circadian function. Abbreviations: AVP: Arginine vasopressin, AII: Angiotensin II, BNST: Bed nucleus of the stria terminalis, CALR: Calretinin, DR, MnR: Dorsal and median raphe nucleus, DMH: Dorsomedial hypothalamic nucleus, ENK: Enkephalin, GABA: *γ*-aminobutyric acid, GHT: Geniculohypothalamic tract, GRP: Gastrin-releasing peptide, IGL: Thalamic intergeniculate leaflet, LHN: Lateral habenula nucleus in the thalamus, mPOA: Medial preoptic area, NMS: Neuromedin S, NPT: Neuropeptide Y, NT: Neurotensin, PVN: Paraventricular nucleus of the hypothalamus, PVT: Paraventricular thalamic nucleus, RHT: Retinohypothalamic tract, SCN: Suprachiasmatic nucleus, VIP: Vasoactive intestinal peptide

**Fig. 4:**
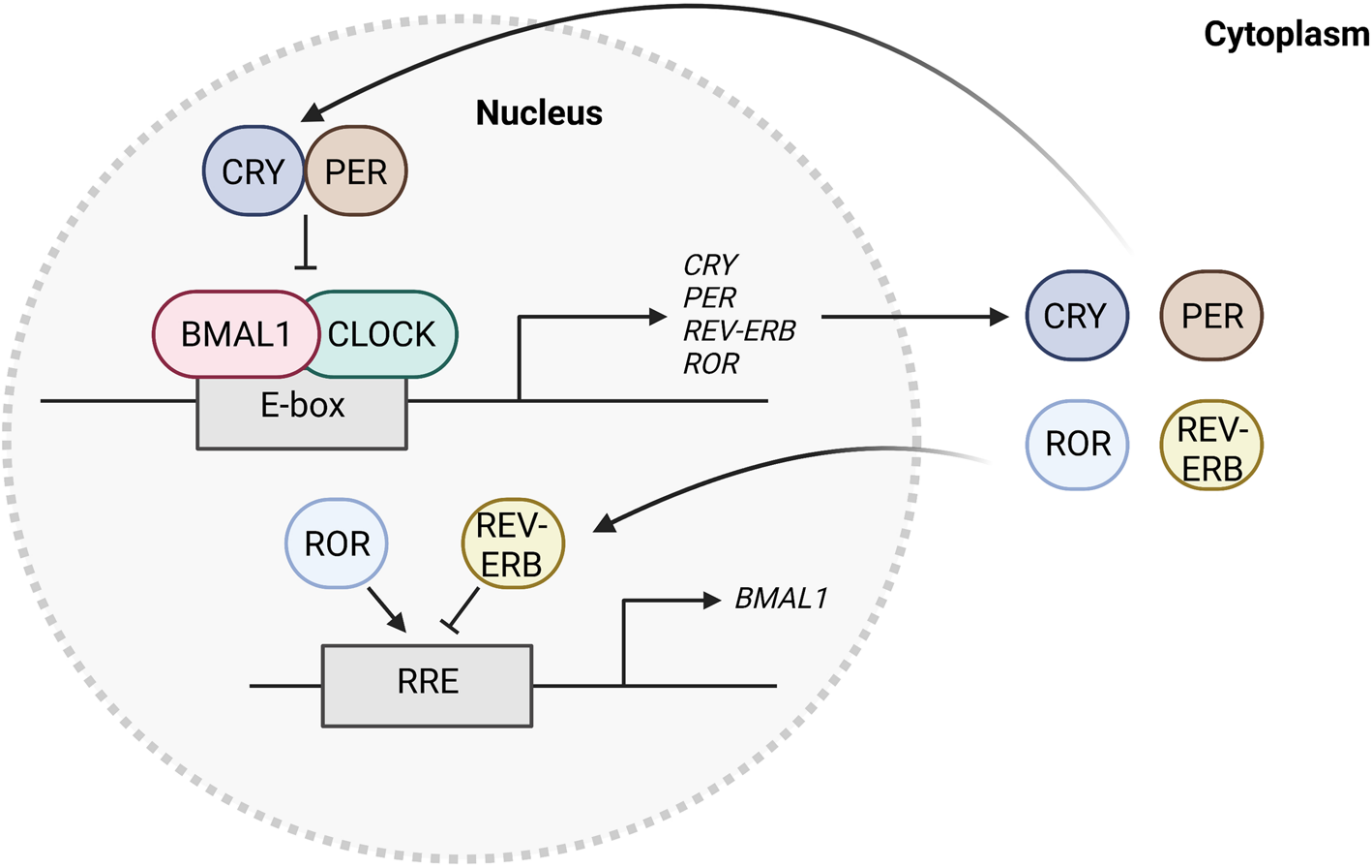
The transcription-translation feedback loop (TTFL), the endogenous molecular circadian clock. The molecular circadian clock operates through the TTFL. The diagram illustrates a simplified representation of the TTFL scheme in human cells capable of exhibiting circadian rhythms. These rhythms are appropriately entrained when the suprachiasmatic nucleus (SCN) is intact, emphasizing its crucial role in maintaining the proper functioning of the circadian system. Abbreviations: BMAL1: Basic helix-loop-helix aryl hydrocarbon receptor nuclear translocator-like 1, CLOCK: Clock circadian regulator, CRY: Cryptochrome, PER: Period, REV-ERB: NR1D1 (Nuclear receptor subfamily 1 group D member 1), ROR: Retinoid-related orphan receptor, RRE: REV-ERB/ROR response element

Circadian regulation is a complex process that involves several elements. The mammalian circadian clock is a hierarchically organized system, coordinated by the bilateral SCN. The role of the SCN in regulating circadian rhythms encompasses three main aspects. First, the SCN network operates as an afferent/efferent neural circuit, directly influencing circadian rhythms through interconnected pathways (Fig. 2). Second, neuromodulatory cells within the SCN modulate the central circadian clock mechanism (Fig. 3). Finally, on a molecular level, individual cells throughout the body possess their own cell-autonomous transcriptional-translational feedback loop (TTFL) involving the transcription of “circadian genes” (Fig. 4). These genes, in turn, activate the transcription of various other genes, with multiple negative and positive feedback loops, all of which are ultimately governed by the SCN. As an impairment in any of these components could lead to circadian disruptions in tauopathies, it is crucial to assess their functionality in these diseases to guide preventive measures and treatment strategies.

### Neuronal circuitry of the suprachiasmatic nucleus

The primary input to the SCN is from the melanopsin-expressing retinal ganglion cells (mRGCs) via the retinohypothalamic tract (RHT). The intergeniculate leaflet (IGL) in the thalamus provides a non-photic direct input signal to the SCN clock-regulating activity through the geniculohypothalamic tract [52, 54]. Finally, the dorsal and median raphe nuclei also provide non-photic direct input to SCN [53, 54]. Next, clock-regulating signals transfer out of the SCN to various regions, including the paraventricular nucleus of the hypothalamus, subparaventricular zone, dorsomedial hypothalamus, medial preoptic area, paraventricular thalamic nucleus, lateral habenula, and the bed nucleus of the stria terminalis [55–59] (Figs. 2, 3).

### The neuromodulatory cell processing in the suprachiasmatic nucleus

The SCN leverages exogenous or zeitgeber cues (light-dark shift, photoperiod, food intake, exercise, body temperature, etc.), a process called entrainment to reset and synchronize the rhythmic expression of the circadian genes [60, 61]. While entrainment is crucial for circadian regulation, the SCN can maintain biological periodicity even under conditions of restricted cue signaling in experimental settings [60]. Nearly all SCN neurons express *γ*-aminobutyric acid (GABA), with specific neuronal groups co-expressing neuropeptides, including arginine vasopressin (AVP), vasoactive intestinal peptide (VIP), gastrin-releasing hormone, neuropeptide Y, enkephalin, neuromedin S, and calretinin. AVP-positive (AVP+) and VIP-positive (VIP+) neurons primarily regulate intercellular circadian rhythms [62] (Fig. 3). Despite its small size, the SCN exhibits a well-defined topographical organization. It consists of a core region enriched with VIP+ neurons, which receives the most afferent signals to the SCN. Surrounding the core is a shell region composed of AVP+ neurons, which transmit efferent circadian information [52, 62]. Single-cell RNA sequencing showed the richness of cell types in the SCN, including astrocytes, microglia, oligodendrocytes, NG2-glia, tanycytes, endothelial cells, and ependymal cells [63, 64]. Recent evidence suggests that non-neuronal cells also contribute to circadian control, which will be further discussed in subsequent paragraphs.

### The transcription-translation feedback loop (TTFL)

The mammalian internal circadian clock is regulated by the endogenous molecular rhythmicity of individual cells through a TTFL [65] (Fig. 4). Briefly, the expression of heterodimeric transcriptional activators, basic helix-loop-helix aryl hydrocarbon receptor nuclear translocator-like 1 (BMAL1) (also known as ARNTL) and clock circadian regulator (CLOCK), which induce the expression of their own negative regulators, period (PER), and cryptochrome (CRY). PER-CRY inhibits the transcriptional activity of BMAL1-CLOCK. When the levels of PER-CRY have sufficiently decreased, BMAL1-CLOCK can reinitiate transcription. The BMAL1-CLOCK complex also activates the expression of nuclear receptors REV-ERBs; also known as nuclear receptor subfamily 1 group D member 1 (NR1D1), which counteract retinoid-related orphan receptor (ROR)-mediated BMAL1 expression. Molecular entrainment machinery also contributes to the timely degradation of circadian-related proteins via post-translational modifications and a degradation-promoting system involving the proteasome and autophagosome, which are also vital regulators of TTFLs.

## Neural and molecular basis of tau-related neuropathology and circadian disruptions in the SCN

The underlying mechanisms of circadian dysfunction in tauopathies are not yet well understood. Limited studies (in number and the type of tauopathy) conducted in humans have primarily focused on counting SCN neurons using quantitative, but also biased methods and screening for abnormal tau accumulation (Table 2). In the case of AD, some of the few studies found a scarce number of diffuse A*β* (*β*-amyloid) plaques [10].

**Table 2:** Studies investigating changes in the human suprachiasmatic nucleus in aging and tauopathies

Tauopathy	Reference	Subjects			Tau inclusion^(^^b)^	Evidence of neuronal death^(^^c)^	Note
		Sex	Age at death (years) (a)	Described diagnosis (Pathologically confirmed)			
AD	Swaab et al. (1985) [9]	M:12 F:16	M: 16–85; mean [SEM]: 46[7] F: 10–93; mean [SEM]: 58[7]	Non-demented	Not tested	Control	Astrocytosis
		M:1 F: 3	Pooled M/F; mean [SEM]: 79[5]	SDAT (Senile dementia of the Alzheimer’s type)	Not tested	* (AVP, number) ** (AVP, volume)	
	Swaab et al. (1992) [14]	M: 4 F: 3	M: 47–85; mean [SEM]: 72[9] F: 59–91; mean [SEM]: 79[10]	Non-demented	Absent	Control	
		M: 5 F: 7	M: 45–97; mean [SEM]: 71[9] F: 56–90; mean [SEM]: 75[5]	Alzheimer’s disease	Mild (only a very few/some)^(b)^	**	
	Zhou et al. (1995) [12]	M: 24 F: 22	M: 26–82; mean [SEM]: 53[3] F: 26–81; mean [SEM]: 53[3]	No primary neurological or psychiatric disease	Not tested	Control	
		M: 11 F: 10	M: 51–84; mean [SEM]: 68[4] F: 59–83; mean [SEM]: 71[3]	Alzheimer’s disease	Not tested	** (VIP, number) ** (VIP, volume)	
	Stopa et al. (1999) [10]	Pooled M/F: 10	Pooled M/F: mean [SEM]: 63[12]	Non-demented	Absent	Control	
		Pooled M/F: 19	Pooled M/F: mean [SEM]: 72[8] (d)	Alzheimer’s disease (Probable; NINCDS-ADRDA)	Rare	*** (AVP)	
	Wu et al. (2007) [13]	M: 11 F: 13	M: 19–83; mean [SEM]: 52[8] F: 21–85; mean [SEM]: 45[5]	Non-demented	Not tested	Control	
		M: 4 F: 7	M: 64–87; mean [SEM]: 71[5] F: 65–82; mean [SEM]: 73[2]	Alzheimer’s disease (Preclinical)	Not tested	NS (AVP/VIP number) NS (AVP/VIP density)	
		M: 4 F: 7	M: 64–83; mean [SEM]: 72[5] F: 59–86; mean [SEM]: 72[4]	Alzheimer’s disease (Late clinical)	Not tested	* (AVP/VIP number) * (AVP/VIP density)	
	Harper et al. (2008) [11]	M: 5 F: 4	Pooled M/F: mean [SEM]: 64[11]	Non-demented	Not tested	Control	
		M: 17–19 (e) F: 0	Pooled M/F: mean [SEM]: 70[7]	Alzheimer’s disease (Pooled early (*Braak* III-IV) and late (*Braak* V-VI; including LBD)	Not tested	** (AVP) *** (NT)	Increased Glia/Neuron ratio
	Wang et al. (2015) [15]	Pooled M/F: 10	Pooled M/F: mean [SD]: 91[6]	Non-demented	Not tested	Pooled subjects and assessed a correlation coefficient between the number of VIP-SCN neurons and circadian actigraphy data (VIP only): R = 0.83 *P* = 0.008	Delayed circadian activity rhythm phase
		Pooled M/F: 7	Pooled M/F: mean [SD]: 90[5]	Alzheimer’s disease	Not tested		
PiD	Harper et al. (2008) [11]	M: 5 F: 4	Pooled M/F: mean[SEM]: 64 11]	Non-demented	Not tested	Control	
		M: 2 (PiD only; (f)) F: 0	Non-mentioned	Pick’s disease	Not tested	** (AVP)	
	Stopa et al. (1999) [10]	Pooled M/F: 13	Pooled M/F: mean[SEM]: 63[12]	Non-demented	Not tested	Control	
		Pooled M/F: 3	Pooled M/F: mean[SEM]: 72[8] (d)	Pick’s disease	Not tested	* (NT)	
CBD	Harper et al. (2008) [11]	M: 5 F: 4	Pooled M/F: mean[SEM]: 64[11]	Non-demented	Not tested	Control	
		M: 2/ F: 0	Non-mentioned	Corticobasal degeneration	Not tested	** (AVP)	
PSP	De Pablo-Fernandes et al. (2018) [21]	Pooled M/F: 5	Pooled M/F: median (IQR): 84 (78–88) (g)	Non-demented	Absent	Control	
		Pooled M/F: 5	Pooled M/F: median (IQR): 74 (70–81) (g)	Progressive supranuclear palsy	2 subjects: Mild 3 subjects: Moderate	Not tested	

(a) Round to integer, (b) As described in the reference, (c) Statistical significance (As described in the reference), (d) Pooled all demented cases in the reference (AD and FTD, etc.), (e) Subjects- 19: total, 18: glia/neuron ratio, 17: AVP, NT data, (f) The number of cases is part of the pooled disease group (FTD; PiD and CBD), (g) Pooled subjects collected for another ROI investigation. Abbreviations: AD: Alzheimer’s disease, AVP: Arginine vasopressin, CBD: Corticobasal degeneration, FTD: Frontotemporal disorders, PiD: Pick’s disease, PSP: Progressive supranuclear palsy, N: the number of subjects (only patients), NT: Neurotensin

### Changes in SCN neuronal and glial numbers in tauopathies (Table 2)

Of all tauopathies, changes in neuronal numbers were investigated in AD and PiD only. Swaab et al. used unbiased stereological methods to quantify SCN neurons in twenty-eight controls and four individuals with pathologically proven late-stage AD. Compared to control individuals in the age group 60–80 years of age which had an average of ~ 50,000 SCN neurons, the late-stage AD cases had an average of ~ 20,000 [9]. Stopa et al. looked at autopsy-verified nineteen AD, three PiD, and ten control cases [10] and found a substantial loss of AVP+ and neurotensin+ neurons (the only two neuronal populations quantified) and astrocytosis in AD and PiD compared to controls. Harper et al. also detected loss of AVP+ and neurotensin neuronal populations in the SCN in AD [11]. Zhou et al. evaluated the number of VIP+ neurons in SCN in normal aging and AD. They found that VIP+ neuronal number drops in males but not females during normal aging [12]. Although there was a trend of a lower number of VIP+ neurons in AD, the results were statistically significant only in females with an early disease onset (age 65 years and younger). Unfortunately, the low number of AD cases and the use of semi-quantitative methods hamper the interpretation of results from this paper. A single study by Wu et al. probed the number of AVP+ and VIP+ neurons (lumped together) and MT1-expressing neurons in controls and individuals at progressive *Braak* stages [13]. They found a decrease in neuronal density of all neuronal populations tested, but only at end-stage cases.

In summary, these few studies highlight the presence of neuronal loss and astrogliosis in the SCN among individuals with AD and PiD, at least at end-disease stages. Concerning the timing of SCN neuronal loss in the early stages of the disease, only one study encompassed individuals at progressive AD stages, implying that SCN neuronal loss tends to occur later in the disease progression. The question about chronology cannot be tested in PiD or the majority of tauopathies as they lack a clear neuropathological staging system. Despite some studies attempting to determine the specific neuronal subtypes more susceptible to damage, none of them delved into the separate examination of both AVP and VIP neuronal populations within the same research investigation, revealing a notable gap in the research.

### Tau inclusions in the human SCN (Table 2)

Of all tauopathies, changes in neuronal numbers were investigated in AD and PSP only. De Pablo-Fernandez et al. performed semi-quantitative analyses (using a scale ranging from absent to very severe) in the SCN of five PSP cases and five control cases. The researchers observed AT8 (phosphorylated tau) immunoreactivity of mild to moderate severity in all PSP cases. However, the researchers did not attempt to quantify neurons [21]. Swaab et al. examined the hypothalamic nuclei of seven control subjects and twelve AD patients using immunohistochemistry against three tau antibodies [14]. Tau changes in SCN neurons were mild compared to other examined nuclei, such as the tuberomammillary nucleus and nucleus tuberalis lateralis, which was surprising given that the same group reported that the number of SCN neurons decreases in AD [14]. Although it may seem paradoxical to have severe cell death in a context of low abnormal protein burden, similar discrepancies have been observed in AD studies focusing on other subcortical nuclei, where the loss of tau signal does not necessarily coincide with the death of neurons [66]. Possibly, the abnormal tau inclusions disappeared due to neuronal death. The few studies probing other nuclei in the anterior hypothalamus in AD suggest that SCN are more vulnerable to tau burden or tau-related neuronal death than neighboring nuclei containing VIP+ and AVP+ neurons. For instance, the supraoptic nucleus remains free of p-tau and A*β* even at late AD stages [14, 67]. The periventricular nucleus accumulates a tau burden similar to the SCN, but neuronal numbers remain stable, even at late AD stages [14, 67].

In conclusion, the limited number of studies imply that neuronal tau inclusions are present in both PSP and AD, with a greater load observed in PSP [68]. It remains uncertain whether the tau inclusions in PSP also encompass astroglial, a significant gap given the association of tufted astrocytes with PSP. Furthermore, the timeline for the emergence of tau inclusions and whether a specific SCN neuronal population is more susceptible to these inclusions remain uncharted territories.

### Changes in TTFL and circadian genes in tauopathies

We could not find any study focusing on TTFL changes in humans with tauopathies, only investigation in experimental models.

These experimental studies suggest that tau-induced changes in the SCN lead to disruption of TTFL molecular pathways. Transgenic mouse models expressing human tau protein have shown dysregulation of the molecular circadian clock in the SCN. The human MAPT-expressing P301S tauopathy mouse PS-19 model shows an aberrant expression of *Bmal1* in the SCN [69]. In another study with transgenic MAPT P301L (Tg4510) mouse model exhibited a disrupted circadian behavior cycle with impaired circadian gene, *Bmal1,* and *Per2* transcriptional oscillation. The same study detected *p*-tau immunoreactivity in the SCN, implying that tauopathy in the SCN can contribute to circadian clock dysregulation at behavioral and molecular levels [70]. Recent research has indicated a connection between pathological tau accumulation in the SCN and disrupted proteostasis, which is thought to contribute to the molecular desynchronization of circadian rhythms by impairing the rhythmic clearance of clock machinery proteins. Chaperone-mediated autophagy (CMA), a process responsible for the targeted degradation of intracellular proteins, has been associated with the molecular restoration of the circadian rhythm [71]. Disruption of the CMA process in the SCN can impact the cycling of clock elements such as BMAL1, CRY1, PER1, and REV-ERBa [72]. CMA plays a role in degrading the drivers of the circadian cycle, namely BMAL1 and CLOCK, and therefore has an important role in circadian molecular feedback loops. BMAL1 and CLOCK interact with heat shock cognate 71-kDa protein (HSC70) and lysosome-associated membrane protein 2 (LAMP2A), components of the CMA-active lysosomal complex. BMAL1 oscillations regulate the expression of heat-shock protein 70 (HSP70), which belongs to the same chaperone group as HSC70 and is associated with tau solubility [69]. These findings suggest that disruption of CMA could serve as the link between tauopathies and the dysregulation of circadian rhythms. The CMA also may regulate the ubiquitin-proteasome system (UPS) [73], which in turn degrades several circadian oscillating proteins [74]. The UPS degrades BMAL1 through the chaperone machinery suppressor of the G2 allele of skp1/heat-shock protein 90 (SGT1/HSP90), which is highly expressed in the SCN [75]. In summary, tau accumulation in SCN can impact the integrity of SCN neurons and contribute to dysregulation of the circadian clock at both local and global levels. However, there is still much to discover regarding the molecular changes associated with the desynchronization of circadian rhythmicity, especially in human tauopathies.

## Astrocytes in the SCN and circadian disruptions in tauopathies

Astrocytes support the neuronal network by forming and pruning synapses, ion-homeostasis of neurons, modulating neurotransmission, contributing to microglial phagocytosis, and modulating neuroinflammation. Astrocytic activity follows circadian patterns. Astrocytes in culture exhibit daily rhythms [76], cytokine expression oscillates in astrocytes [77], and core clock genes such as *Dbp*, *Nr1d1,* and *Fabp7* show higher amplitude in mouse astrocytes than in neurons [64]. Also, astrocytes are abundant in human SCN [10] and recent evidence suggests that astrocytic function influences circadian function [78]. In a study with mouse SCN explants, Patton et al. genetically ablated *Bmal1* from SCN astrocytes and determined that astrocytes can initiate SCN rhythms and lengthen the circadian period, albeit less efficiently than neurons [79]. although astrocytes did not influence the circadian phase [79]. Another study showed similar results: lengthened clock gene expression in SCN and locomotion after selective ablation of *Bmal1* from SCN astrocytes [80]. Astrocytes also contribute to entraining neuronal circadian rhythm in the SCN. For instance, the SCN-dense population of glial fibrillary acidic protein (GFAP) positive astrocytes show diurnal activity [81], which directly modulates circadian cycles of extracellular glutamate and GABA to inhibit SCN neurons [82–84]. Also, astrocytes may inhibit AVP + -neuronal activity by eliminating extracellular glutamate in the SCN shell for fine-tuning circadian clock synchrony [83]. Other studies indicate that astrocytes, specifically a subpopulation expressing a specific transcription factor (doublecortin-like), stimulate AVP+ neurons [85]. These apparent contradictions (activating vs. inhibiting AVP+ neurons) support the view that astrocytic subpopulations perform different roles similarly to neuronal subpopulations (*i.e.*, excitatory, and inhibitory neurons).

Although the number of studies investigating astrocytic contribution to circadian dysregulation, and *vice-versa*, in tauopathies, is meager, even in experimental models, some recent papers suggest potential links. Other studies suggested that astrocytic clock dysfunction exacerbates AD pathology since this disruption affects astrocytic activity, such as inflammatory response, and makes neurons more vulnerable to insults [86, 87]. A few other studies examined the relationship between circadian genes and brain amyloidosis. Deletion of core genes *Bmal1* and *Clock* suppresses the expression of *Chi3l1* in astrocytes leading to increased A*β* accumulation [88]. Finally, astrocytic circadian deficits may lead to sleep disruption, exacerbating AD progression. For instance, *Fabp7* mutations lead to sleep fragmentation [89]. *Fabp7* is strongly expressed in astrocytes, and its expression is controlled by the core clock in astrocytes [90]. Experimental studies provide compelling evidence of a connection between tau pathology, astrocytic dysfunction, and disruptions in molecular mechanisms that regulate circadian rhythms and SCN integrity. However, only a handful of studies investigated astrogliosis in the SCN of AD and PiD cases (Table 2), but even a fine mapping of astrocytic changes in any tauopathies is yet to be done. An area of research interest is what is causing circadian dysfunction in astrocytes in AD since tau deposits in astrocytes are not a prominent AD feature (and A*β* deposits are extracellular). A strategy to investigate the indirect effect of tau pathology in astrocytes in AD on the desynchronization of the circadian clock and the contribution of tau astrogliopathy to sleep-wake disturbances in humans is to include in addition to a regular control group, a comparison group of tauopathy with prominent tau inclusions in astrocytes, such as PSP, CBD, or ARTAG. Contemporary technology can facilitate human studies in this regard. For instance, in-situ proteomics and transcriptomics can be instrumental in understanding the nature of astrocytic alterations within the SCN in the context of tauopathies. Single-cell technology studies can precisely identify changes in the expression of circadian genes in specific astrocytic subpopulations within both affected and unaffected brain regions in different tauopathies.

## Microglia in the SCN and circadian disruptions in tauopathies

Microglia play a crucial role in primary immune responses, modulating neuroinflammation, reactive oxygen processing, and protein phagocytosis. Evidence shows that an intrinsic circadian clock regulates microglia activity [91, 92] and displays diurnal morphological change in SCN and subcortical wake-sleep-promoting nuclei [93]. Therefore, circadian abnormalities may lead to microglia dysfunction, indirectly exacerbating tauopathies. A few papers interrogated whether primary microglia dysfunction can lead to circadian abnormalities. Microglia-mediated cytokines recruit other activated microglia and stimulate other microglial and astrocytic transcription in the SCN [94]. Two recent papers show contradicting results concerning microglial ablation as a cause of abnormal circadian rhythms [95, 96]. Unfortunately, only a limited number of papers have explored the interplay between circadian function, microglia, and the modulation of tauopathies, and these studies have primarily been conducted in experimental models. Nevertheless, these studies indicate that microglial dysfunction occurs within the SCN, even at early stages of the disease. In a mouse model of tauopathy (P301L), characterized by abnormal clock gene expression in the SCN, microglial activation precedes the accumulation of abnormal tau inclusions [97]. Given the paucity of studies focusing on the relationship between microglia-circadian function and tau abnormalities, in a context of the increased recognition of microglia in modulating tauopathies, examining the microglial population and morphology in progressive tauopathies may be the next step in understanding the role of microglia in the progression of tauopathies and their impact on the master circadian rhythms mediated by the SCN.

## *β*-Amyloid pathology and circadian disruptions in the SCN

Although this review focuses on tau pathology, it is impossible to ignore that AD, the most well-known study of tauopathy regarding circadian dysfunction also features prominent A*β* pathology that may contribute to circadian dysregulation. Therefore, investigations of the SCN in AD must include studies of A*β* pathology. Interestingly, the SCN is practically devoid of A*β* pathology, even at late AD stages [10], even when several hypothalamic nuclei accumulate plaques. Still, experimental models suggest that amyloidosis impacts circadian rhythmicity even if A*β* pathology is lacking in the SCN, which brings clues for future human studies. For instance, a study in 5xFAD mice demonstrated altered expression patterns of the circadian clock genes *Bmal1* and *Per2* in the SCN and circadian behavior [98]. Indeed, another study in 5xFAD mice shows a neuronal loss in SCN and one of its primary afferents, IGL, already at four months old, preceding severe cognitive impairment [99]. However, there was a lack of evidence of A*β* (and tau) accumulation in the SCN. A third study found that human APP-expressing Tg-SwDI mice feature a shortened free-running period and dampening of day/night differential excitability of SCN neurons [100].

Collectively, these studies in humans and mice suggest that A*β* pathology is more globally associated with networks interacting with SCN as opposed to directly causing SCN degeneration. Interestingly, human amyloid overexpress model mouse with AD-pathogenesis triggering factor; TREM2 showed greater A*β* burden and microglial reactivity in the hippocampal formation [101], suggesting amyloidosis can deteriorate sleep-wake-circadian disorder globally. A*β* related degeneration of mRGCs, another primary SCN afferent, may contribute to circadian dysfunction [102, 103]. RGC loss is a prominent feature of glaucoma, prevalent in individuals with circadian disorders and AD [104]. One investigation of postmortem human retinas reported A*β* immunoreactivity in the ganglion cell layer [101]. Another study detected A*β* accumulation inside and around mRGCs using optical coherence tomography, and A*β* burden correlated with sleep efficienc [103, 105] . However, it is unclear if RGC accumulate A*β* pathology in humans or if their activities are impacted by A*β* retinal deposits leading to activating specific inflammatory and neurodegenerative processes and downregulated mitochondrial and photoreceptor-related pathways [106]. Interestingly, although one study showed positive tau immunoreactivity in specific ganglion cells [102], most studies probing the retina for tau deposits in AD, CBD, and PSP failed to find pathological tau in the ganglion cells at the RHT [105–107]. Altogether, these studies indicate the need to include A*β* pathology metrics in SCN and interconnected structures as part of studies investing the basis of circadian dysfunction in AD. The plausible hypothesis is that comparing cases with AD-tau to those without amyloid deposition may provide insights into the role of amyloid. Although we were unable to find specific literature, evaluating neuronal loss, pathological changes in the SCN, and circadian dysfunctions, particularly in tauopathies without amyloidopathies as compared to AD-tauopathies, could serve as an informative approach to address the role of amyloid in circadian rhythm.

## Conclusion and future directions

This review aimed to provide an in-depth overview of the current understanding regarding the vulnerability of the SCN to tauopathies and its contribution to circadian dysregulation and sleep-wake disturbances, and vice-versa. Still, despite the compelling evidence indicating the presence of abnormal tau inclusions in the SCN and the occurrence of circadian dysfunction in various tauopathies, there is a notable scarcity of detailed neuropathological studies on regional and systemic molecular changes associated with SCN susceptibility to tau pathology, particularly in humans. Therefore, we suggest a research strategy, founded on fundamental questions that *remain unanswered*, including:

1. *At what stage of the disease, do tau inclusions start accumulating in the SCN in conditions such as AD and other tauopathies?*

The first part of this question requires the collection of brain tissue from cases at progressive disease stages, which can be achieved in some tauopathies such as AD [108], PSP [109], and CTE [110]. Including cases at progressive stages is an approach to model disease progression in a cross-sectional design, which is inherent in postmortem studies of human brain. Unfortunately, most of the other tauopathies lack a well-defined staging system due to the lack of antemortem biomarkers and scarcity of early-stage cases in autopsy cohorts. Once cohorts of progressive cases are in place, neuropathological methods can help understand the chronology of changes (tau accumulation, neuronal loss, evidence of neuroinflammation, and glial changes).

2. *Which specific subtypes of SCN cells are most susceptible to the diverse range of tauopathies? What factors contribute to the selective vulnerability of SCN neurons to tauopathies like AD, PSP, PiD, and others?*

This question can be better answered using neuropathological methods. In this approach, SCN slides from cases with pathologically confirmed tauopathies undergo multi-labeled staining for cell and pathological markers, which can be quantified using stereological methods to avoid counting biases. Given the tiny size of SCN, using in-situ transcriptomics combined with tau staining to detect affected cells, may inform the most important cell types for subsequent stereological assessment. Once the most vulnerable SCN cells for each tauopathy are identified, the next step is to investigate the molecular signature of these vulnerable cells vs. resilient cells to identify factors underlying vulnerability. Single-nucleus RNA sequencing may provide answers to this question.

3. *Does tau-associated pathology in the SCN correlate with different circadian abnormalities?*

While there have been significant technological advancements in measuring circadian rhythms in humans, such as using actigraphy to detect changes in the period, phase, and amplitude of rest/active brain activity, or methods to assess indirect metrics of circadian integrity like automatic physical responses (e.g*.*, body temperature, heart rate, and respiratory rate) and hormone levels in plasma/saliva (e.g*.*, melatonin and corticotropin-releasing hormone), there is currently no available technology for in vivo measurement of SCN integrity in humans. As so, circadian dysfunction does not necessarily indicate SCN pathology because the problem may be derived from other parts of the circadian control system. An attempt to address this question would require long-term studies involving cohorts of individuals who are periodically assessed for circadian dysfunction until as close as possible to their death, at which point their brains can be examined directly to evaluate the SCN and interconnected regions, and the clinical and pathological data can be compared.

4. *What is the role of neuroinflammation in tau deposition and SCN degeneration in tauopathies?*

Neuroinflammation has emerged as a double-edged sword in the pathogenesis of neurodegenerative diseases, with the potential to either exacerbate the disease or protect the brain against the deleterious effects of the pathological process [111]. Therefore, it is critical to determine whether and when the SCN develops a neuroinflammatory pattern in diverse tauopathies, ideally through the examination of cases at progressive stages of the disease. Equally important is the need to identify whether neuroinflammation is prompted by tau deposition or another mechanism. Examining the sequence of events between these two processes may provide valuable insights.

5. *Most tauopathies feature tau accumulation in astroglia, such as PSP and CBD. Astrocytes are known to contribute to circadian regulation. Does tau astrogliopathy worsen circadian dysfunction that is caused by SCN degeneration?*

Answering this question is not straightforward when using human brains. One approach could involve employing high-resolution in-situ transcriptomics to examine the expression of circadian genes in astrocytes with and without tau accumulation. However, even if the results reveal distinct profiles, they can only indirectly suggest whether tau astrogliopathy is influencing circadian regulation or merely causing localized dysfunction. An important unresolved query is whether tau pathology affects SCN astroglia. In conditions like PSP, for instance, while tufted astrocytes are a pathological hallmark, they are much more prevalent in cortical regions than in subcortical areas, where neuronal inclusions predominate [68].

6. *Is there any indication of a lack of coordination in the TTFL in tauopathies? If so, does this lack of coordination result from tau pathology in cells that are susceptible to tau pathology in the brain, or does it stem from the inability of a dysfunctional SCN to centrally regulate circadian function, or perhaps a combination of both factors?*

Experiments with mouse models indicate that dysfunction in the SCN leads to a general lack of coordination in the TTFL. It is conceivable that tau deposition might impair a cell’s ability to express TTFL components too. In experimental models is possible to knock out specific genes in specific brain areas to understand the impact of this change. This kind of genetic manipulation is not possible in humans, but by conducting a detailed analysis of SCN changes in progressive stages of tauopathies in parallel with mapping molecular changes in circadian genes within the SCN and related areas in the same cases, preferentially with single nucleus technology, may enable drawing inferences regarding this question.

In summary, addressing these questions is of utmost importance in understanding the intricate interplay between tauopathies, SCN degeneration, and circadian dysfunction [112–114]. Establishing whether disrupted circadian function is a cause, or a consequence of abnormal tau accumulation represents a significant gap in our understanding. This knowledge is vital for developing targeted interventions to prevent and manage circadian dysregulation and sleep disturbances in individuals affected by tauopathies.

## Data Availability

Data sharing does not apply to this article as no datasets were generated or analyzed during the current study.

## Abbreviations

3R/4R: a combination of three and four microtubule-binding repeats
3R: three microtubule-binding repeats
4R: four microtubule-binding repeats
AD: Alzheimer’s disease
AGD: Argyrophilic grain disease
ARNTL: Aryl hydrocarbon receptor nuclear translocator-like 1
ARTAG: Aging-related tau astrogliopathy
AVP: Arginine vasopressin
AVP +: Arginine vasopressin-positive
A*β*: *β*-amyloid
BMAL1: Basic helix-loop-helix aryl hydrocarbon receptor nuclear translocator-like 1
CBD: Corticobasal degeneration
CLOCK: Clock circadian regulator
CMA: Chaperone-mediated autophagy
CRY: Cryptochrome
CTE: Chronic traumatic encephalopathy
GABA: *γ*-aminobutyric acid
GFAP: Glial fibrillary acidic protein
GGT: Globular glial tauopathy
HSC70: Heat shock cognate 71-kDa protein
HSP70: Heat-shock protein 70
IGL: Intergeniculate leaflet
LAMP2A: Lysosome-associated membrane protein 2
MAPT: Microtubule-associated protein tau
mRGCs: Melanopsin-expressing retinal ganglion cells
NR1D1: Nuclear receptor subfamily 1 group D member 1
NREM: Non-rapid eye movement
PART: Primary age-related tauopathy
*p*-tau: Hyperphosphorylated tau
PER: Period
PiD: Pick’s disease
PSP: Progressive supranuclear palsy
REM: Rapid eye movement
RHT: Retinohypothalamic tract
ROR: Retinoid-related orphan receptor
SCN: Suprachiasmatic nucleus
SGT1/HSP90: skp1/heat-shock protein 90
SWS: Slow wave sleep
TTFL: Transcriptional-translational feedback loops
UPS: Ubiquitin-proteasome system
VIP: Vasoactive intestinal peptide
VIP +: Vasoactive intestinal peptide-positive

## References

[1] Pollak CP, Perlick D (1991). Sleep problems and institutionalization of the elderly. J Geriatr Psychiatry Neurol..

[2] Spitznagel MB, Tremont G, Davis JD, Foster SM (2006). Psychosocial predictors of dementia caregiver desire to institutionalize: caregiver, care recipient, and family relationship factors. J Geriatr Psychiatry Neurol..

[3] Kovacs GG, Kovacs GG, Alafuzoff I (2018). Chapter 25 - Tauopathies. Handbook of clinical neurology [internet].

[4] Kovacs GG, Ghetti B, Goedert M (2022). Classification of diseases with accumulation of tau protein. Neuropathol Appl Neurobiol..

[5] Crary JF, Trojanowski JQ, Schneider JA, Abisambra JF, Abner EL, Alafuzoff I (2014). Primary age-related tauopathy (PART): a common pathology associated with human aging. Acta Neuropathol..

[6] Duyckaerts C, Braak H, Brion J-P, Buée L, Del Tredici K, Goedert M (2015). PART is part of Alzheimers disease. Acta Neuropathol..

[7] Kovacs GG (2019). Molecular pathology of neurodegenerative diseases: principles and practice. J Clin Pathol..

[8] Deboer T (2018). Sleep homeostasis and the circadian clock: do the circadian pacemaker and the sleep homeostat influence each other’s functioning?. Neurobiol Sleep Circadian Rhythms..

[9] Swaab DF, Fliers E, Partiman TS (1985). The suprachiasmatic nucleus of the human brain in relation to sex, age and senile dementia. Brain Res..

[10] Stopa EG, Volicer L, Kuo-Leblanc V, Harper D, Lathi D, Tate B (1999). Pathologic evaluation of the human Suprachiasmatic nucleus in severe dementia. J Neuropathol Exp Neurol..

[11] Harper DG, Stopa EG, Kuo-Leblanc V, McKee AC, Asayama K, Volicer L (2008). Dorsomedial SCN neuronal subpopulations subserve different functions in human dementia. Brain..

[12] Zhou J-N, Hofman MA, Swaab DF (1995). VIP neurons in the human SCN in relation to sex, age, and Alzheimer’s disease. Neurobiol Aging..

[13] Wu Y-H, Zhou J-N, Van Heerikhuize J, Jockers R, Swaab DF (2007). Decreased MT1 melatonin receptor expression in the suprachiasmatic nucleus in aging and Alzheimer’s disease. Neurobiol Aging..

[14] Swaab DF, Grundke-Iqbal I, Iqbal K, Kremer HPH, Ravid R, van de Nes JAP (1992). τ and ubiquitin in the human hypothalamus in aging and Alzheimer’s disease. Brain Res..

[15] Wang JL, Lim AS, Chiang W-Y, Hsieh W-H, Lo M-T, Schneider JA (2015). Suprachiasmatic neuron numbers and rest-activity circadian rhythms in older humans: SCN and rest-activity rhythms. Ann Neurol..

[16] Walker KR, Tesco G (2013). Molecular mechanisms of cognitive dysfunction following traumatic brain injury. Front Aging Neurosci..

[17] Boone DR, Sell SL, Micci M-A, Crookshanks JM, Parsley M, Uchida T (2012). Traumatic Brain Injury-Induced Dysregulation of the Circadian Clock. Lyons LC, editor. PLoS ONE..

[18] Yamakawa GR, Brady RD, Sun M, McDonald SJ, Shultz SR, Mychasiuk R (2020). The interaction of the circadian and immune system: Desynchrony as a pathological outcome to traumatic brain injury. Neurobiol Sleep Circadian Rhythms..

[19] Brunetti V, Testani E, Iorio R, Frisullo G, Luigetti M, Di Giuda D (2016). Post-encephalitic parkinsonism and sleep disorder responsive to immunological treatment: a case report. Clin EEG Neurosci..

[20] Bluett B, Pantelyat AY, Litvan I, Ali F, Apetauerova D, Bega D, et al. Best practices in the clinical Management of Progressive Supranuclear Palsy and Corticobasal Syndrome: a consensus statement of the CurePSP centers of care. Front Neurol [Internet]. 2021; [cited 2023 May 17]; 12. Available from: 10.3389/fneur.2021.694872 . 10.3389/fneur.2021.694872 PMC8284317 34276544

[21] De Pablo-Fernández E, Courtney R, Warner TT, Holton JL (2018). A histologic study of the circadian system in Parkinson disease, multiple system atrophy, and progressive Supranuclear palsy. JAMA Neurology..

[22] Lin W, Lin Y-K, Yang F-C, Chung C-H, Hu J-M, Tsao C-H (2023). Risk of neurodegenerative diseases in patients with sleep disorders: a nationwide population-based case-control study. Sleep Med..

[23] Gemignani A, Pietrini P, Murrell JR, Glazier BS, Zolo P, Guazzelli M (2005). Slow wave and rem sleep mechanisms are differently altered in hereditary pick disease associated with the TAU G389R mutation. Arch Ital Biol..

[24] Walsh CM, Ruoff L, Walker K, Emery A, Varbel J, Karageorgiou E (2017). Sleepless night and day, the plight of progressive Supranuclear palsy. Sleep..

[25] Peter-Derex L, Yammine P, Bastuji H, Croisile B (2015). Sleep and Alzheimer’s disease. Sleep Med Rev..

[26] Leng Y, Musiek ES, Hu K, Cappuccio FP, Yaffe K (2019). Association between circadian rhythms and neurodegenerative diseases. Lancet Neurol..

[27] Aldrich MS, Foster NL, White RF, Bluemlein L, Ba GP (1989). Sleep abnormalities in progressive supranuclear palsy. Ann Neurol..

[28] Oh JY, Walsh CM, Ranasinghe K, Mladinov M, Pereira FL, Petersen C (2022). Subcortical neuronal correlates of sleep in neurodegenerative diseases. JAMA Neurol..

[29] Prinz PN, Peskind ER, Vitaliano PP, Raskind MA, Eisdorfer C, Zemcuznikov N (1982). Changes in the sleep and waking EEGs of nondemented and demented elderly subjects. J Am Geriatr Soc..

[30] Petit D, Gagnon J-F, Fantini ML, Ferini-Strambi L, Montplaisir J (2004). Sleep and quantitative EEG in neurodegenerative disorders. J Psychosom Res..

[31] Cooke JR, Ancoli-Israel S (2011). Normal and abnormal sleep in the elderly. Handb Clin Neurol..

[32] Van Cauter E, Leproult R, Plat L (2000). Age-related changes in slow wave sleep and REM sleep and relationship with growth hormone and cortisol levels in healthy men. JAMA..

[33] Falgàs N, Walsh CM, Yack L, Simon AJ, Allen IE, Kramer JH, et al. Alzheimer’s disease phenotypes show different sleep architecture. Alzheimers Dement. 2023;alz.12963. 10.1002/alz.12963 PMC10404632 36749893

[34] Arand D, Bonnet M, Hurwitz T, Mitler M, Rosa R, Sangal RB (2005). The clinical use of the MSLT and MWT. Sleep..

[35] Littner MR, Kushida C, Wise M, Davila DG, Morgenthaler T, Lee-Chiong T (2005). Practice parameters for clinical use of the multiple sleep latency test and the maintenance of wakefulness test. Sleep..

[36] Cooper AD, Josephs KA (2009). Photophobia, visual hallucinations, and REM sleep behavior disorder in progressive supranuclear palsy and corticobasal degeneration: a prospective study. Parkinsonism Relat Disord..

[37] Lee SE, Rabinovici GD, Mayo MC, Wilson SM, Seeley WW, DeArmond SJ (2011). Clinicopathological correlations in corticobasal degeneration. Ann Neurol..

[38] Oh J, Eser RA, Ehrenberg AJ, Morales D, Petersen C, Kudlacek J (2019). Profound degeneration of wake-promoting neurons in Alzheimer’s disease. Alzheimers Dement..

[39] Adams JW, Alosco ML, Mez J, Alvarez VE, Huber BR, Tripodis Y (2020). Association of probable REM sleep behavior disorder with pathology and years of contact sports play in chronic traumatic encephalopathy. Acta Neuropathol..

[40] Saper CB, Fuller PM (2017). Wake–sleep circuitry: an overview. Curr Opin Neurobiol..

[41] Lew CH, Petersen C, Neylan TC, Grinberg LT (2021). Tau-driven degeneration of sleep- and wake-regulating neurons in Alzheimer’s disease. Sleep Med Rev..

[42] Volicer L, Harper DG, Manning BC, Goldstein R, Satlin A (2001). Sundowning and circadian rhythms in Alzheimer’s disease. AJP..

[43] Okawa M, Mishima K, Hishikawa Y, Hozumi S, Hori H, Takahashi K (1991). Circadian rhythm disorders in sleep-waking and body temperature in elderly patients with dementia and their treatment. Sleep..

[44] Hatfield CF, Herbert J, van Someren EJW, Hodges JR, Hastings MH (2004). Disrupted daily activity/rest cycles in relation to daily cortisol rhythms of home-dwelling patients with early Alzheimer’s dementia. Brain..

[45] Homolak J, Mudrovčić M, Vukić B, Toljan K (2018). Circadian rhythm and Alzheimer’s disease. Med Sci (Basel)..

[46] Winer JR, Morehouse A, Fenton L, Harrison TM, Ayangma L, Reed M (2021). Tau and β-amyloid burden predict Actigraphy-measured and self-reported impairment and misperception of human sleep. J Neurosci..

[47] Walsh CM, Ruoff L, Varbel J, Walker K, Grinberg LT, Boxer AL (2016). Rest-activity rhythm disruption in progressive supranuclear palsy. Sleep Med..

[48] Suzuki K, Miyamoto T, Miyamoto M, Hirata K (2009). The core body temperature rhythm is altered in progressive supranuclear palsy. Clin Auton Res..

[49] Schmidt C, Berg D, Herting PS, Junghanns S, Schweitzer K (2009). Loss of nocturnal blood pressure fall in various extrapyramidal syndromes. Mov Disord..

[50] Blessing EM, Parekh A, Betensky RA, Babb J, Saba N, Debure L (2022). Association between lower body temperature and increased tau pathology in cognitively normal older adults. Neurobiol Dis..

[51] Maywood ES, Chesham JE, Winsky-Sommerer R, Hastings MH (2021). Restoring the molecular clockwork within the Suprachiasmatic hypothalamus of an otherwise Clockless mouse enables circadian phasing and stabilization of sleep-wake cycles and reverses memory deficits. J Neurosci..

[52] Todd WD, Venner A, Anaclet C, Broadhurst RY, De Luca R, Bandaru SS (2020). Suprachiasmatic VIP neurons are required for normal circadian rhythmicity and comprised of molecularly distinct subpopulations. Nat Commun..

[53] Hay-Schmidt A, Vrang N, Larsen PJ, Mikkelsen JD (2003). Projections from the raphe nuclei to the suprachiasmatic nucleus of the rat. J Chem Neuroanat..

[54] Meyer-Bernstein EL, Morin LP. Differential serotonergic innervation of the Suprachiasmatic nucleus and the lntergeniculate leaflet and its role in circadian rhythm modulation. J Neurosci. 1996;16. 10.1523/JNEUROSCI.16-06-02097.1996 PMC6578502 8604054

[55] Cermakian N, Waddington Lamont E, Boudreau P, Boivin DB (2011). Circadian clock gene expression in brain regions of Alzheimer ‘s disease patients and control subjects. J Biol Rhythm..

[56] Dai J, Swaab DF, Van Der Vliet J, Buijs RM (1998). Postmortem tracing reveals the organization of hypothalamic projections of the suprachiasmatic nucleus in the human brain. J Comp Neurol..

[57] Simerly RB, Swanson LW (1986). The organization of neural inputs to the medial preoptic nucleus of the rat. J Comp Neurol..

[58] Watts AG, Swanson LW, Sanchez-Watts G (1987). Efferent projections of the suprachiasmatic nucleus: I. Studies using anterograde transport of Phaseolus vulgaris leucoagglutinin in the rat. J Comp Neurol..

[59] Buijs RM (1978). Intra- and extrahypothalamic vasopressin and oxytocin pathways in the rat. Cell Tissue Res..

[60] Aschoff J (1960). Exogenous and endogenous components in circadian rhythms. Cold Spring Harb Symp Quant Biol..

[61] Vitaterna MH, Takahashi JS, Turek FW (2001). Overview of circadian rhythms. Alcohol Res Health..

[62] Ono D, Honma KI, Honma S (2021). Roles of neuropeptides, VIP and AVP, in the mammalian central circadian clock. Front Neurosci..

[63] Xu P, Berto S, Kulkarni A, Jeong B, Joseph C, Cox KH (2021). NPAS4 regulates the transcriptional response of the suprachiasmatic nucleus to light and circadian behavior. Neuron..

[64] Wen S, Ma D, Zhao M, Xie L, Wu Q, Gou L (2020). Spatiotemporal single-cell analysis of gene expression in the mouse suprachiasmatic nucleus. Nat Neurosci..

[65] Patke A, Young MW, Axelrod S (2020). Molecular mechanisms and physiological importance of circadian rhythms. Nat Rev Mol Cell Biol..

[66] Eser RA, Ehrenberg AJ, Petersen C, Dunlop S, Mejia MB, Suemoto CK (2018). Selective vulnerability of brainstem nuclei in distinct Tauopathies: a postmortem study. J Neuropathol Exp Neurol..

[67] Diodati D, Cyn-Ang L, Kertesz A, Finger E (2012). Pathologic evaluation of the Supraoptic and paraventricular nuclei in dementia. Can J Neurol Sci..

[68] Roemer SF, Grinberg LT, Crary JF, Seeley WW, McKee AC, Kovacs GG (2022). Rainwater Charitable Foundation criteria for the neuropathologic diagnosis of progressive supranuclear palsy. Acta Neuropathol..

[69] Han SM, Jang YJ, Kim EY, Park SA (2022). The change in circadian rhythms in P301S transgenic mice is linked to variability in Hsp70-related tau disaggregation. Exp Neurobiol..

[70] Stevanovic K, Yunus A, Joly-Amado A, Gordon M, Morgan D, Gulick D (2017). Disruption of normal circadian clock function in a mouse model of tauopathy. Exp Neurol..

[71] Dice JF (2007). Chaperone-mediated autophagy. Autophagy..

[72] Juste YR, Kaushik S, Bourdenx M, Aflakpui R, Bandyopadhyay S, Garcia F (2021). Reciprocal regulation of chaperone-mediated autophagy and the circadian clock. Nat Cell Biol..

[73] Xia Y (2022). Role of Ubiquilin-2 in Proteostasis and tau aggregation in Tauopathies. J Neurosci..

[74] Busino L, Bassermann F, Maiolica A, Lee C, Nolan PM, Godinho SIH (2007). SCF ^Fbxl3^ controls the oscillation of the circadian clock by directing the degradation of Cryptochrome proteins. Science..

[75] Lu R, Dong Y, Li JD (2020). Necdin regulates BMAL1 stability and circadian clock through SGT1-HSP90 chaperone machinery. Nucleic Acids Res..

[76] Prolo LM, Takahashi JS, Herzog ED (2005). Circadian rhythm generation and entrainment in astrocytes. J Neurosci..

[77] Hight K, Hallett H, Churchill L, De A, Boucher A, Krueger JM (2010). Time of day differences in the number of cytokine-, neurotrophin- and NeuN-immunoreactive cells in the rat somatosensory or visual cortex. Brain Res..

[78] Hastings MH, Brancaccio M, Gonzalez-Aponte MF, Herzog ED. Circadian rhythms and astrocytes: the good, the bad, and the ugly. Annu Rev Neurosci. 2023;46 null. 10.1146/annurev-neuro-100322-112249 PMC10381027 36854316

[79] Patton AP, Smyllie NJ, Chesham JE, Hastings MH (2022). Astrocytes sustain circadian oscillation and Bidirectionally determine circadian period, but do not regulate circadian phase in the Suprachiasmatic nucleus. J Neurosci..

[80] Tso CF, Simon T, Greenlaw AC, Puri T, Mieda M, Herzog ED (2017). Astrocytes regulate daily rhythms in the suprachiasmatic nucleus and behavior. Curr Biol..

[81] Leone MJ, Marpegan L, Bekinschtein TA, Costas MA, Golombek DA (2006). Suprachiasmatic astrocytes as an interface for immune-circadian signalling. J Neurosci Res..

[82] Brancaccio M, Edwards MD, Patton AP, Smyllie NJ, Chesham JE, Maywood ES (2019). Cell-autonomous clock of astrocytes drives circadian behavior in mammals. Science..

[83] Brancaccio M, Patton AP, Chesham JE, Maywood ES, Hastings MH (2017). Astrocytes control circadian timekeeping in the Suprachiasmatic nucleus via glutamatergic signaling. Neuron..

[84] Patton AP, Morris EL, McManus D, Wang H, Li Y, Chin JW (2023). Astrocytic control of extracellular GABA drives circadian timekeeping in the suprachiasmatic nucleus. Proc Natl Acad Sci..

[85] Coomans C, Saaltink D-J, Deboer T, Tersteeg M, Lanooij S, Schneider AF (2021). Doublecortin-like expressing astrocytes of the suprachiasmatic nucleus are implicated in the biosynthesis of vasopressin and influences circadian rhythms. Glia..

[86] Costa R, Montagnese S (2021). The role of astrocytes in generating circadian rhythmicity in health and disease. J Neurochem..

[87] McKee CA, Lananna BV, Musiek ES (2020). Circadian regulation of astrocyte function: implications for Alzheimer’s disease. Cell Mol Life Sci..

[88] Lananna BV, McKee CA, King MW, Del-Aguila JL, Dimitry JM, Farias FHG, et al. Chi3l1/YKL-40 is controlled by the astrocyte circadian clock and regulates neuroinflammation and Alzheimer’s disease pathogenesis. Sci Transl Med. 2020;12. 10.1126/scitranslmed.aax3519 PMC7808313 33328329

[89] Gerstner JR, Perron IJ, Riedy SM, Yoshikawa T, Kadotani H, Owada Y (2017). Normal sleep requires the astrocyte brain-type fatty acid binding protein FABP7. Sci Adv..

[90] Gerstner JR, Bremer QZ, Vander Heyden WM, Lavaute TM, Yin JC, Landry CF (2008). Brain fatty acid binding protein (Fabp7) is diurnally regulated in astrocytes and hippocampal granule cell precursors in adult rodent brain. PLoS One..

[91] Muthukumarasamy I, Buel SM, Hurley JM, Dordick JS. NOX2 inhibition enables retention of the circadian clock in BV2 microglia and primary macrophages. Front Immunol [Internet]. 2023; [cited 2023 Apr 3];14. Available from: 10.3389/fimmu.2023.1106515 . 10.3389/fimmu.2023.1106515 PMC9939898 36814920

[92] Fonken LK, Frank MG, Kitt MM, Barrientos RM, Watkins LR, Maier SF (2015). Microglia inflammatory responses are controlled by an intrinsic circadian clock. Brain Behav Immun..

[93] Liu H, Wang X, Chen L, Chen L, Tsirka SE, Ge S (2021). Microglia modulate stable wakefulness via the thalamic reticular nucleus in mice. Nat Commun..

[94] Deng X-H, Bertini G, Palomba M, Xu Y-Z, Bonaconsa M, Nygård M (2010). Glial transcripts and immune-challenged glia in the Suprachiasmatic nucleus of Young and aged mice. Chronobiol Int..

[95] Matsui F, Yamaguchi ST, Kobayashi R, Ito S, Nagashima S, Zhou Z (2023). Ablation of microglia does not alter circadian rhythm of locomotor activity. Mol Brain..

[96] Sominsky L, Dangel T, Malik S, De Luca SN, Singewald N, Spencer SJ (2021). Microglial ablation in rats disrupts the circadian system. FASEB J..

[97] Yoshiyama Y, Higuchi M, Zhang B, Huang S-M, Iwata N, Saido TC (2007). Synapse loss and microglial activation precede tangles in a P301S Tauopathy mouse model. Neuron..

[98] Song H, Moon M, Choe HK, Han D-H, Jang C, Kim A (2015). Aβ-induced degradation of BMAL1 and CBP leads to circadian rhythm disruption in Alzheimer’s disease. Mol Neurodegener..

[99] Nam Y, Kim S, Kim J, Hoe H-S, Moon M (2022). Mesoscopic mapping of visual pathway in a female 5XFAD mouse model of Alzheimer’s disease. Cells..

[100] Paul JR, Munir HA, van Groen T, Gamble KL (2018). Behavioral and SCN neurophysiological disruption in the Tg-SwDI mouse model of Alzheimer’s disease. Neurobiol Dis..

[101] Parhizkar S, Gent G, Chen Y, Rensing N, Gratuze M, Strout G (2023). Sleep deprivation exacerbates microglial reactivity and Aβ deposition in a TREM2-dependent manner in mice. Sci Transl Med..

[102] den Haan J, Morrema THJ, Verbraak FD, de Boer JF, Scheltens P, Rozemuller AJ (2018). Amyloid-beta and phosphorylated tau in post-mortem Alzheimer’s disease retinas. Acta Neuropathol Commun..

[103] La Morgia C, Ross-Cisneros FN, Koronyo Y, Hannibal J, Gallassi R, Cantalupo G (2016). Melanopsin retinal ganglion cell loss in A lzheimer disease. Ann Neurol..

[104] Gubin D, Weinert D (2022). Melatonin, circadian rhythms and glaucoma: current perspective. Neural Regen Res..

[105] Hart de Ruyter FJ, Morrema THJ, den Haan J, Twisk JWR, de Boer JF, Scheltens P (2023). Phosphorylated tau in the retina correlates with tau pathology in the brain in Alzheimer’s disease and primary tauopathies. Acta Neuropathol..

[106] Koronyo Y, Rentsendorj A, Mirzaei N, Regis GC, Sheyn J, Shi H (2023). Retinal pathological features and proteome signatures of Alzheimer’s disease. Acta Neuropathol..

[107] Schön C, Hoffmann NA, Ochs SM, Burgold S, Filser S, Steinbach S (2012). Long-term in vivo imaging of Fibrillar tau in the retina of P301S transgenic mice. PLoS One..

[108] Montine TJ, Phelps CH, Beach TG, Bigio EH, Cairns NJ, Dickson DW (2012). National Institute on Aging-Alzheimer’s association guidelines for the neuropathologic assessment of Alzheimer’s disease: a practical approach. Acta Neuropathol..

[109] Kovacs GG, Lukic MJ, Irwin DJ, Arzberger T, Respondek G, Lee EB (2020). Distribution patterns of tau pathology in progressive supranuclear palsy. Acta Neuropathol..

[110] Mckee AC, Abdolmohammadi B, Stein TD (2018). The neuropathology of chronic traumatic encephalopathy. Handb Clin Neurol..

[111] Wyss-Coray T, Mucke L (2002). Inflammation in neurodegenerative disease--a double-edged sword. Neuron..

[112] Wang C, Holtzman DM (2020). Bidirectional relationship between sleep and Alzheimer’s disease: role of amyloid, tau, and other factors. Neuropsychopharmacol..

[113] Musiek ES, Xiong DD, Holtzman DM (2015). Sleep, circadian rhythms, and the pathogenesis of Alzheimer disease. Exp Mol Med..

[114] Hoyt KR, Obrietan K (2022). Circadian clocks, cognition, and Alzheimer’s disease: synaptic mechanisms, signaling effectors, and chronotherapeutics. Mol Neurodegener..

